# Notes on Tuberculosis1Read before the Virginia State Veterinary Medical Association, Charlottesville, Virginia, January 3, 1895.

**Published:** 1895-02

**Authors:** E. P. Niles

**Affiliations:** Blacksburg, Virginia


### NOTES ON TUBERCULOSIS.[1]

BY E. P. NILES, D.V.M.,
BLACKSBURG, VIRGINIA.

BEFORE introducing my subject for discussion, let us inquire into the true standing of the veterinarian and endeavor to obtain a proper definition for the term. Until a few years back the veterinarian was almost unknown in the United States, and consequently had no standing politically, legally, or socially. Why the veterinarian has not enjoyed the same standing in every respect that his close relative, the physician, has, is simply because the proper definition has not been applied to the term veterinarian by the masses of people. The true veterinarian received, and does yet to a certain extent, a "hard name" the moment he entered college, which requires years of trials and tribulations to live down.

The question naturally arises: What is a proper definition for the word veterinarian? Webster gives as a definition for the word "one skilled in the diseases of cattle or domestic animals." I would say a veterinarian is one who is proficient in all subjects pertaining to veterinary science and has completed the prescribed course in a regularly organized veterinary col-

[1] Read before the Virginia State Veterinary Medical Association, Charlottesville, Virginia, January 3, 1895.

lege. Such men will command the same respect that is due any professional man; and I may add that such men are rapidly taking the place of the ancient "horse doctors," whose character had been, in the main, such as to give to the veterinarian the unjust reputation which has been so hard to live down, and which, to a great extent, still exists. The question may be asked, What bearing does the "horse doctor" have on the veterinarian? Simply this: by the majority of people we were and are yet, to a great extent, classed as horse doctors. It is almost an everyday occurrence for the veterinary practitioner to be addressed with: "Are you the horse doctor?" or "cow doctor" by some ignorant party. The time is now at hand that the veterinarian should command due respect from all classes of people, for he is rapidly placing himself in a position to be classed among the public benefactors in the way of suppressing and controlling contagious diseases among the lower animals, and indirectly among the human family. The hearty co-operation of the physician, however, is needed in this work, and, I am happy to say, our cousins are not slow to lend us their aid. I believe that the day is not far distant when the two professions will march hand in hand against the dreaded contagious diseases, many of which affect man and beast alike. In some of these diseases it is first necessary to control them in the lower animals before they can be controlled in man. In this work the two schools of medicine alone cannot succeed. The co-operation of the State Legislature is needed. The organization of an official body of scientific workers must be effected. Money must be provided for its use, for without the proper funds nothing can be done. The present Board of Health is not even allowed money with which to buy its postage stamps. How can the Board be expected to accomplish any good results under such circumstances?

The most dangerous contagious disease that the veterinary and medical professions have to deal with at the present time is *tuberculosis*. I say it is the most dangerous of all the contagious diseases, because it affects both man and beast alike, and because it is so frequently transmitted from the lower animals to man.

This, until recently, has been one of the hardest diseases known to properly diagnose in all of its stages; especially is this true in relation to the lower animals. It is hardly necessary to state here, however, that we now have a reliable and positive aid to the diagnosis of the disease in all of its stages, *tuberculin*, the *modus operandi* of which does not require a description at this time, for the subject of my last essay is still fresh in your memory. For the introduction of this agent our thanks are due Dr. Koch, of Berlin. For the good results of its use in this country and the practical conclusions drawn from such, all, or nearly, all credit is due the veterinarians. As has frequently been stated, we now have a means at hand by which tuberculosis can be practically stamped out both among the human family and the lower animals. With this end in view the veterinary and the medical professions are working together; yet there is room for them to draw still closer together. They should mingle together in each other's societies, thereby enabling them more effectually to co-operate with each other. When this is done the importance of thorough and careful legislation for the suppression and control of contagious diseases will at once thrust itself upon our legislators. The two professions combined will carry with them much more powerful influence than we can hope to carry alone, especially while our numbers are so small.

The question of legislation for the purpose of stamping out tuberculosis is an important one. Already some of the States in the Union have enacted laws for the purpose, while a few of the States are quarantining against each other, and others are quarantining all herds known to have tuberculous animals among them, without any authority than the general law controlling such contagious and infectious diseases. What will result from such action on the part of the State Board of Health and the State veterinarian remains to be seen. The effect of one State quarantining against another is almost to ruin the cattle industry in the quarantined district. Such a state of affairs exists between Vermont and Massachusetts. The latter has a law purporting to control tuberculosis, but its workings are anything but satisfactory. The law provides for the appointment of inspectors, but does not say that they shall be veterinarians, nor, in fact, medical men of any kind. The consequence is that incompetent men are appointed, more for their political value than anything else. Many of them, I am told, do not know a tubercle from a piece of normal tissue, neither do they know anything about the tuberculin test. They examine in a general way suspected herds, and pronounce them healthy or diseased, according to their best common judgment. Qualified men are condemned for doing their duty, and ignorant men are put in their places. The compensation for animals destroyed amounts to little or nothing; consequently, the dissatisfaction is great.

Legislation on the subject should not be gone into hastily, as evidently has been the case in some of our sister States. Such legislation is bound to meet with opposition and finally defeat. All sides of the question must be carefully weighed, and provision made for any point that is likely to arise in the workings of the law.

Our own State should not be the last to legislate on the subject of this loathsome disease. Our next Legislature should, in some way, be made to see the importance of stamping out the disease, and induced to organize the State Board of Health properly, and give them power and money to act in the matter. To procure the necessary laws it will require the combined effort of our society, with the medical society of this State, as well as our own individual efforts. It is also necessary that the breeders of the State should join us in our efforts.

As I have stated in a previous article, all dairy and breeding herds should be put through the tuberculin test. It is also advisable to make the tests as far as possible on all private herds, and the diseased cattle wherever found destroyed. Such animals, of course, must be paid for by the State. If the owners, however, could be made to look at the matter in the proper light they would see that it would in the end be much more profitable to destroy all diseased animals at once, even though they received nothing for them. By keeping one diseased animal in the herd the disease is constantly being communicated to the other animals, until finally the whole herd may become affected. Besides the danger of communicating the disease to himself and family would at once be avoided by the destruction of the diseased animal or animals. It may be argued that the State cannot afford to pay for these diseased cattle. If the State has not now got the money at hand to carry out the work a slight increase of taxes would readily supply the funds, and no one would, or ought to, object to having his tax only slightly increased if he knew that it was for the purpose of making his herds more valuable and almost entirely cutting off his chance to become a victim to tuberculosis. It would seem, however, that extra taxation is entirely unnecessary, for the amount required for the work would grow less after the first year or two.

The following figures show the approximate cost of exterminating the disease: In 1892 the estimated number of cattle in Virginia was 406,137. Supposing the number has remained stationary, and estimating their value at $15 per head, the total value is $6,104,055. If 2 per cent. of these cattle are tuberculous we then have 8138 tuberculous cattle in Virginia. These cattle in health represent $122,070. Each one of these diseased cattle is capable of communicating the disease to one or more healthy individuals; in fact, one animal may infect a whole herd. But suppose the infection is in the ratio of one to two of every diseased animal each year. We will suppose, then, that in the year 1894 we have in Virginia 8138 tuberculous animals; at the above ratio of infection we will have in 1895 10,850 tuberculous animals; in 1896 we will have 14,466. It will be seen that the number of tuberculous animals is rapidly on the increase, even with this small ratio of infection. If the true ratio of infection could be gotten at, however, I am of the opinion that it would be much greater. What other disease can produce such appalling figures? The infection does not stop with animals of the same species, but may extend to nearly all warm-blooded animals, and especially to the human family. I have stated in a previous article that 50 per cent. of the cases of tuberculosis in the human family come from the bovine species. If one person for every ten tuberculous cattle becomes infected from that source each year, we will have 813 more cases of tuberculosis in the human family in 1895 than in the year previous, and 1085 in 1896, and so on year after year, to say nothing of the spread of the disease by those so infected.

Let us see what would be saved to the State and the private stock owners each year by stamping out the disease.

Let us suppose that one-half of the tuberculous animals are destroyed at once. We would have left but 4069 diseased animals to spread the disease. The number infected by these, according to the above ratio, would be 1356, making a total number of 5425 affected animals in 1895, as compared with 10,850. In 1895 we will slaughter the remaining half of the original number, thus reducing the number of diseased animals to 1356. In 1896 the infection will thereby be reduced to 452 animals, so that in the third year of our work we will have but 1808 animals to slaughter, and at the same time we have gotten rid of the principal source of infection in this short time, thus increasing the value of our herds almost, if not quite, twofold, and greatly lessened the danger to human life. In the first year we have saved the State, by preventing further infection, $20,340. In the second year the amount saved will reach $81,375. In the third year the amount saved will reach the surprisingly large sum of $199,870. It will also be seen that the number of cases of tuberculosis in the human family will be greatly diminished.

Now, suppose the State pays $10 per head for each animal slaughtered. The sum required would be $99,460, averaging about $33,000 per year for the first three years, after which $10,000 per year would carry on the work of the State Board of Health very nicely.

It has been shown above that the actual amount saved over and above the amount expended for the destruction of these animals is $203,125, to say nothing of the human lives that are annually saved.

This is a question that concerns all classes of people alike, and the State can no longer afford to defer action on the matter. The disease is assuming alarming proportions, and unless something is soon done tuberculosis or consumption will be almost as common as common colds.

I have endeavored to show that the veterinarian is now in a position to demand a hearing on these subjects that so constantly menace the public health, and that prompt action on the part of our next Legislature will do much to advance the public welfare and the cattle industry of our State.

